# Compact VHF/UHF Ultrawideband Discone Antenna with Consistent Pattern

**DOI:** 10.3390/s24186147

**Published:** 2024-09-23

**Authors:** Guang Li, Fushun Zhang, Bingnan Wang

**Affiliations:** 1 National Key Laboratory of Microwave Imaging Technology, Chinese Academy of Sciences Aerospace Information Research Institute, Beijing 100090, China; wbn@mail.ie.ac.cn; 2 National Key Laboratory of Science and Technology on Antennas and Microwaves, Xidian University, Xi’an 710071, China; fshzhang@mail.xidian.edu.cn

**Keywords:** very high frequency (VHF), ultra high frequency (UHF), ultrawideband antenna, discone antenna, consistent pattern

## Abstract

A compact VHF/UHF ultrawideband discone antenna with consistent patterns is proposed in this article. The proposed antenna consists of a disk, a modified cone, an inverted cone, four shorting probes, and two sleeves. To improve the radiation angular distortion at high frequencies, two sleeves are inserted into the discone antenna. Higher-order modes are suppressed, and ultrawideband consistent patterns are obtained without antenna size increasing. An inverted cone and four shorting probes are introduced to achieve broadband and profile reduction. An antenna prototype is fabricated and measured. The proposed antenna possesses consistent patterns in a 11.36:1 bandwidth. The pattern nulls is improved by 26.1 dB. The antenna occupies a cylindrical volume of 0.227 $\lambda_{0}$ (D) and 0.096 $\lambda_{0}$ (H). It is a competitive candidate for future in-vehicle communication systems.

## 1. Introduction

The very high frequency (VHF) and ultra high frequency (UHF) communications enable the establishment of vehicle-to-vehicle links over hundreds of kilometers without satellite or land-based infrastructure [1,2,3,4,5,6]. Monopole whip antennas in conjunction with automatic antenna tuners are the primary options for such applications [7,8]. Whip antennas have high profiles and narrow bandwidths. The antennas with low visual signatures is of paramount importance in military communications systems [9]. Ultrawideband communication systems are robust to interference, but the whip antennas limit the utility of such systems. In the ultrawide operating band, as the frequency increases, the angular attenuation of the radiation patterns severely affects the communication performance in the fixed directions. The consistent pattern design is essential for communication systems [10]. Therefore, the development of compact VHF/UHF antennas with consistent vertical polarization and omnidirectional radiation characteristics in a broad bandwidth is urgently required [11].

Contrast to monocone antennas, discone antennas have more potential to achieve ultrawideband characteristics [12]. The research methodology of monocone antennas can be applied to discone antennas. Various studies on cone shape optimization have been reported [13]. Pear-like cones [14], quadratic wire-shaped cones [15], and curvature-shaped skeletal cones [16] are optimized to obtain wideband characteristics. An elliptical-shaped disk and a bell-shaped ground plane generate a 2-D planar discone with a 28.57:1 bandwidth [17]. And some new processes have also been applied to discone antennas, such as three-dimensional (3D) printing technology [18]. These discone antennas possess wideband characteristics but do not consider the angular distortion of radiation patterns in a compact size [19], which is critical for vehicle communication systems.

For asymmetric discone antennas, it is a challenge to obtain consistent low-angular distortion patterns without degrading other electrical characteristics. In this paper, we present a compact VHF/UHF ultrawideband discone antenna based on comprehensive theoretical analysis of the classical discone antenna far field modes. By introduction of two sleeves, the nulls of the radiation pattern at high frequencies are filled to −5.5 dB [20]. The discone antenna has consistent patterns over the 11.36:1 bandwidth.

## 2. Antenna Design

### 2.1. Antenna Structure

**Figure 1:**
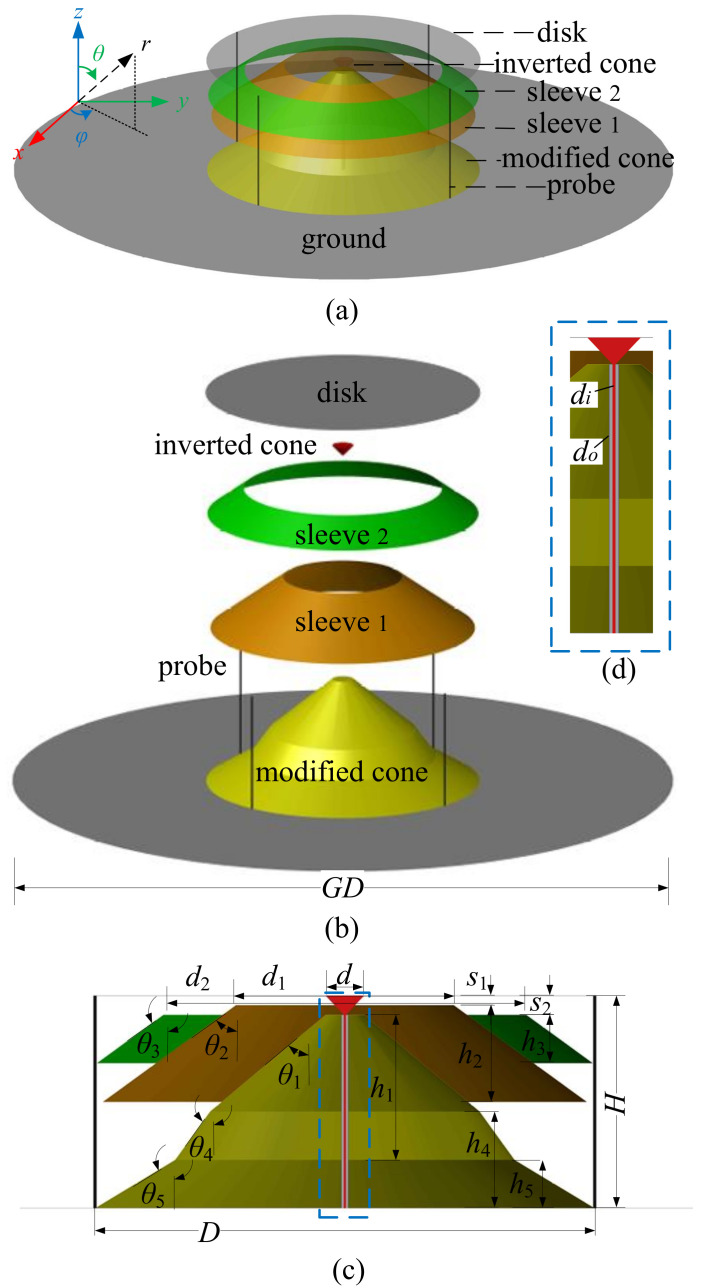
Antenna structure. (**a**) perspective view; (**b**) explosive view; (**c**) sectional view; (**d**) feeding structure.

As shown in Figure 1a, the proposed antenna is composed of a disk, a modified cone, an inverted cone, four shorting probes, and two sleeves. To show the antenna structure more clearly, an exploded view is shown in Figure 1b. Based on a discone antenna, four shorting probes evenly distributed on the disk circumference with an interval of 90° are inserted to electrically connect the cone to the disk. This lengthens the current path, which motivates the low-frequency mode of operation and equivalently reduces the antenna profile [21]. To achieve good impedance matching, an inverted cone is introduced at the coaxial cable connection. The inverted cone has a diameter of *d* at the base and a height of $s_{2}$. The two sleeves are located in the region between the disk and the cones, as shown in the sectional view shown in Figure 1c. The distances from the disk are $s_{1}$ and $s_{2}$ for sleeve 1 and sleeve 2, respectively. This structural arrangement allows the proposed antenna to improve the pattern distortion without increasing in size. The proposed antenna is fed by a coaxial line that is connected to the inverted cone on the inside and to the modified cone on the outside shown in Figure 1d. The outer and inner diameters of the coaxial line are $d_{o}$ and $d_{i}$, respectively. The overall size of the proposed antenna is ϕ 310 mm × 132 mm, and the radius of the ground plane is 375 mm. We use the trust region framework in the time domain solver for parameter optimization. The optimized structure parameters of the proposed antenna are listed in Table 1.

### 2.2. Radiation Analysis

**Figure 2:**
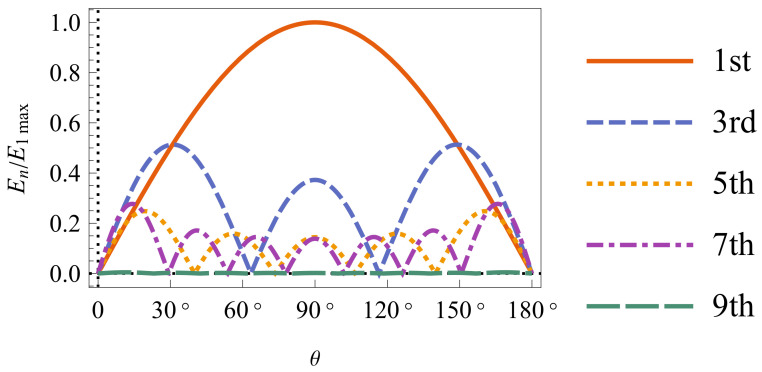
Mode electric fields in E-plane with ka=5.6, $\theta_{0}=54^{\circ }$.

A discone antenna can be regarded as an asymmetric biconical antenna with a disk and a cone. The spatial region is divided into the antenna region (I) and the radiation region (II). The electric field is an infinite series of eigenfunctions, and transverse magnetic (TM) modes exist in the radiation region. The higher-order modes of the discone antenna are produced as operating frequency increases [22]. To analyze the effect of different modes on the radiation pattern, the normalized electric fields of different modes in the E-plane are shown in Figure 2. With antenna length ka of 5.6 and cone angle $\theta_{0}$ of $54^{\circ }$, the mode field is calculated in Wolfram Mathematica. The 1st-order mode is dominant and the pattern is sinusoidally distributed. The normalized amplitudes of 3rd, 5th, and 7th-order modes are 0.51, 0.25, and 0.27. The 9th-order mode approaches zero. The 3rd, 5th, and 7th-order modes have 2, 4, and 6 nulls, respectively. The total field is the summation of all mode fields. The discone antenna is monopole-like at low frequency, and the 1st-order mode is dominant. At high frequency, the null of the higher order mode will cause the total field distortion in the E-plane [19]. To obtain consistent patterns at an ultrawide operating band, the higher-order modes at high frequencies should be suppressed. The discone operates as a monopole with the 1st-order mode when the higher-order modes are fully suppressed. In this article, small sleeves are proposed to suppress the higher-order modes of the discone antenna. The effect of two sleeves on the far-field patterns of the discone antenna is investigated as follows.

**Table 1:** Antenna Structure Parameters.

Parameter	*H*	*D*	$h_{1}$	$h_{2}$	$h_{3}$	$h_{4}$	$h_{5}$	*d*
Value (mm)	132	310	120	60	30	60	30	24
Parameter	$d_{1}$	$d_{2}$	$d_{i}$	$d_{o}$	$s_{1}$	$s_{2}$	GD	
Value (mm)	135	235	1.3	4.1	6	12	375	
Parameter	$\theta_{1}$	$\theta_{2}$	$\theta_{3}$	$\theta_{4}$	$\theta_{5}$			
Value (°)	50	54	54	46	59			

**Figure 3:**
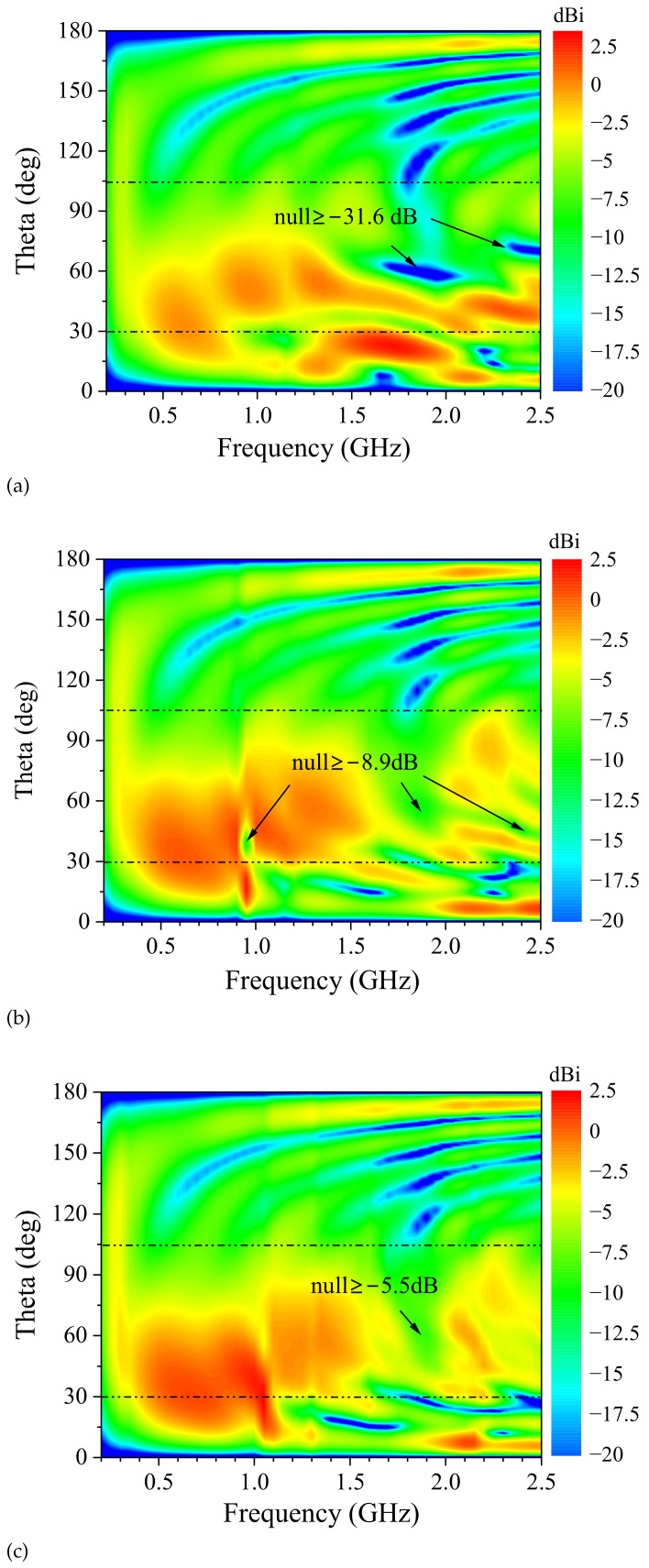
E-plane patterns of (**a**) without sleeves; (**b**) with sleeve 1; (**c**) proposed antenna.

The radiation patterns in the H-plane are omnidirectional due to the symmetric design of 90° rotation around the *z*-axis. The E-plane patterns are improved by introducing two small sleeves. To illustrate the effects of the two sleeves on the radiation pattern, the E-plane patterns of without sleeves, with sleeve 1, and the proposed antenna are shown in Figure 3.

As shown in Figure 3a, the discone antenna without introducing sleeves has a monopole-like pattern over the frequency range of 220 MHz to 1.2 GHz. As frequency increases, the maximum beam points to around 25°. The pattern nulls are produced around 35° at 1.2 GHz to 2.0 GHz and around 60° at 1.6 GHz to 2.05 GHz. When the frequency increases to 2.35 GHz, the pattern null is around 70°. The major pattern nulls are −31.6 dB in 55° at 1.8 GHz, −18.9 dB in 65° at 2.4 GHz, and −18.6 dB in 59° at 1.6 GHz. The pattern splits across from 1.2 GHz to 2.5 GHz, leading to a decrease of −31.6 dB in gain, which seriously affects the performance of the discone antenna in communication systems.

To enhance the antenna directivity in the broadside, the pattern with sleeve 1 are shown in Figure 3b. The structure parameters of sleeve 1 are consistent with Figure 1c. The pattern depression in broadside changes from −31.6 dB in Figure 3a to −8.6 dB in Figure 3b, and the null region is reduced at frequencies above 1.6 GHz. The pattern null of 1.8 GHz is improved from −31.6 dB to −5.7 dB. The pattern null of 2.35 GHz is −8.9 dB, and an additional null emerges at 0.95 GHz. The worst pattern null is improved to −8.9 dB. This verifies that sleeve 1 improves the pattern depression, especially around 1.8 GHz.

To further improve the antenna pattern higher than 1.6 GHz, the sleeve 2 is inserted into the antenna structure. The pattern is shown in Figure 3c. The pattern null near 45° is eliminated at frequencies above 2 GHz. The pattern null at 0.95 GHz is also eliminated, and the pattern depression in broadside is compressed to the region of from 1.7 GHz to 2 GHz. The worst pattern null in Figure 3c is −5.5 dB in 58° at 1.9 GHz. Comparing radiation patterns in Figure 3a and in Figure 3c, the area of pattern depression is significantly compressed. With two sleeves insertion, the worst pattern null is improved by 26.1 dB from −31.6 dB to −5.5 dB.

### 2.3. Operating Mechanism

**Figure 4:**
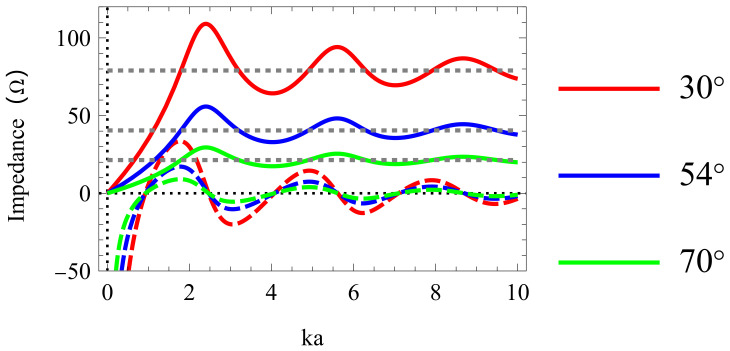
Input impedance of proposed antenna (dash lines represent corresponding characteristic impedance).

The impedance characteristics of the discone antenna with the cone angle $\theta_{0}$ of $30^{\circ }$, $54^{\circ }$, $70^{\circ }$ are shown in Figure 4. The input resistance curves rise from zero and oscillate around the characteristic impedance $Z_{0}$ in the ka range of from 0 to 10. The input reactance curves start with a large negative value and oscillate, decaying around zero ohm. The characteristic impedance $Z_{0}$ decreases, and the impedance bandwidth will be broadened as the flare angle $\theta_{0}$ increases. However, the antenna profile will deteriorate. The $\theta_{0}$ is set to $54^{\circ }$ considering the impedance bandwidth and antenna profile.

To illustrate the pattern improvement in principle, the electromagnetic field distributions of the design progress are studied. As shown in Figure 3a, the pattern nulls are distributed above 1.2 GHz, concentrating in the frequency ranges of 1.6 GHz to 2.05 GHz and 2.35 GHz to 2.5 GHz. To briefly analyze the effect of two sleeves on the field distributions, the electric and magnetic field distributions of without sleeves, with sleeve 1 and the proposed antenna are given at 1.6 GHz and 2.5 GHz.

**Figure 5:**
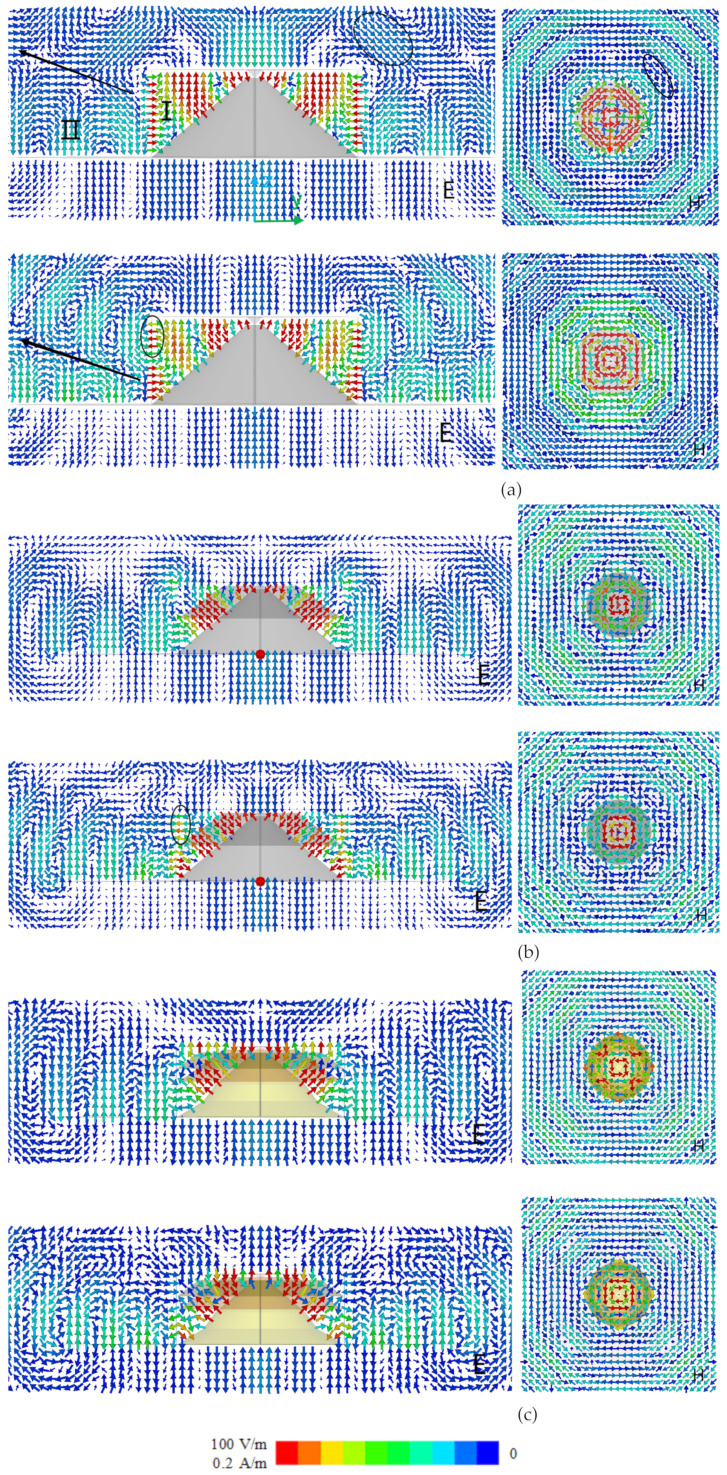
E- and H-fields of (**a**) without sleeves; (**b**) with sleeve 1; (**c**) proposed antenna at 1.6 GHz and 2.5 GHz.

The field from region I passes through the boundary and into region II, forming electromagnetic radiation. As shown in Figure 5a, the electric and magnetic fields propagate outward in a corrugated manner and are distributed between the disk and the cone in region I and mainly along the ground in region II. Higher-order modes are produced in region II, caused by the distributed fields in region I. Higher-order modes affect the field distribution, and there is a pattern depression in the specified direction at 1.6 GHz and 2.5 GHz. This is the basic principle that produces the pattern nulls around the broadside in the far field.

The field distributions in region II are determined by the field in region I using the boundary conditions. The sleeve 1 is introduced into the discone antenna to change the electric field distribution in region I. As shown in Figure 5b, the electric field at 1.6 GHz is compressed to the region between sleeve 1 and the cone. Compared with electric field at 1.6 GHz in Figure 5a, the higher-order mode is suppressed. But the higher-order mode still exists at 2.5 GHz because the electric field distribution in region I is not changed. This shows that sleeve 1 modifies the electric field distribution around 1.6 GHz in the near field and fills the pattern null at 1.6 GHz in the far field.

As shown in Figure 5b, the higher-order mode still produces pattern nulls at 2.5 GHz. The sleeve 2 is introduced to change the fields around 2.5 GHz in region I and suppress the higher-order mode in region II, resulting in pattern null filling in the far field. As shown in Figure 5c, the electric field is *∞*-shaped in the E-plane and the magnetic field is omnidirectional in the H-plane. The suppression of the electric field at the boundary in Figure 5c suggests that the introduction of sleeve 1 suppresses the higher-order modes at 2.5 GHz. With the introduction of two sleeves, the proposed antenna achieves a monopole-like field distribution in the broadside at the high frequency.

### 2.4. Parametric Sweep

The analysis results show that sleeve 1 fills the null in the frequency range of 1.6 GHz to 2.0 GHz, and sleeve 2 improves the pattern of 2.1 GHz to 2.5 GHz as shown in Figure 3. Considering the impedance characteristics and the volume constraint, the flare angle of both sleeves is set to $54^{\circ }$. The design parameters for the two sleeves are the sleeve heights and diameters, including $h_{2}$, $h_{3}$, $d_{1}$, and $d_{2}$. The E-plane patterns sweep with geometric parameters of sleeve 1 and sleeve 2 for the optimal design. We illustrate the pattern variation with $h_{2}$ and $d_{1}$ in the band of 1.6 GHz to 2.0 GHz and that with $h_{3}$ and $d_{2}$ in the band of 2.1 GHz to 2.5 GHz.

**Figure 6:**
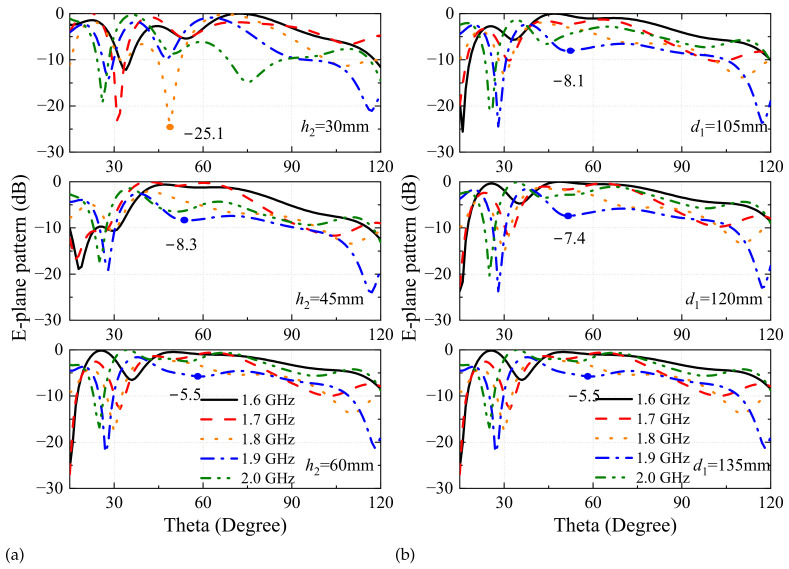

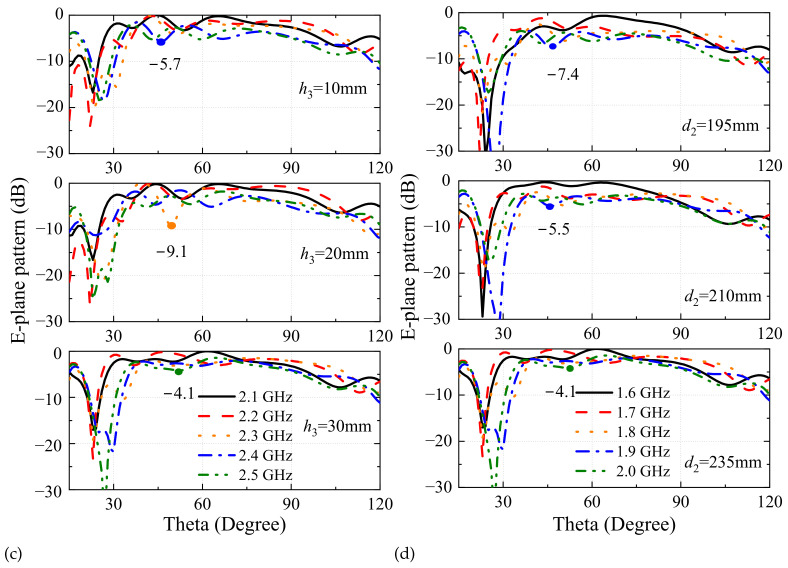
Normalized E-plane pattern with different (**a**) $h_{2}$; (**b**) $d_{1}$; (**c**) $h_{3}$; (**d**) $d_{2}$.

Figure 6a shows the normalized E-plane pattern variation with $h_{2}$. When $h_{2}$ is 30 mm, the pattern has a worst null of −25.1 dB at 1.8 GHz. When $h_{2}$ is increased to 45 mm, the pattern nulls are filled in the band of 1.6 GHz to 1.7 GHz, but pattern depression occurs in the frequency range from 1.8 GHz to 2.0 GHz. The worst depression is −8.3 dB at 1.9 GHz. The pattern null is improved across the band of 1.6 GHz to 2.0 GHz when $h_{2}$ is increased to 60 mm, especially at 2.0 GHz, but pattern null at 1.9 GHz still reaches −5.5 dB. As shown in Figure 6b, the worst null is varied from −8.1 dB to −5.5 dB, with $d_{1}$ varying from 105 mm to 135 mm. The variation verifies the improvement of sleeve 1 on the antenna pattern. Sleeve 1 will exceed the discone volume boundary when $d_{1}$ becomes larger.

Figure 6c investigates the normalized E-plane pattern varying with $h_{3}$ in the 2.1 GHz to 2.5 GHz band. When $h_{3}$ is 10 mm, the worst pattern null occurs at 2.4 GHz with a value of −5.7 dB. When $h_{3}$ is increased to 20 mm, the worst pattern null is shifted to 2.3 GHz, and its value deteriorates to −9.7 dB. As $h_{3}$ increases to 30 mm, the null value is optimized to −4.1 dB, located at 2.5 GHz. Sleeve 2 will exceed the discone volume limit with $h_{3}$ increasing. As $d_{2}$ increases from 195 mm to 235 mm, the pattern nulls are from −7.4 dB to −4.1 dB as Figure 6d shows. The gain decreasing around the 30° is consistent with the pattern variation as Figure 3 shows.

## 3. Measurement and Comparison

**Figure 7:**
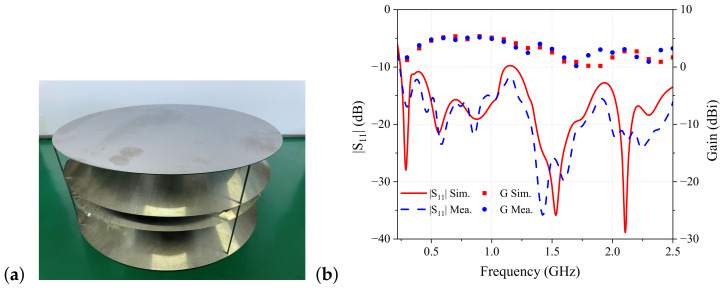
(**a**) Antenna prototype; (**b**) measured $|S_{11}|$ and gain.

A prototype of the proposed antenna is fabricated as shown in Figure 7a. The disk, cone, inverted cone, and sleeve cones are machined from metal and connected to the coaxial line by welding to form the antenna prototype. The reflection coefficient of the antenna prototype is measured using a vector network analyzer. The radiation pattern is measured using an antenna near-field test system in an anechoic chamber.

As shown in Figure 7b, the measured reflection coefficients agree well with the simulated results. The reflection coefficients are less than −10 dB in the operating frequency of from 220 MHz to 2.5 GHz. In vehicle communication system applications, the communication direction is along the broadside. The maximum gain is observed in the broadside of θ ranging from 30° to 105°. The variation of gain with frequency is shown in Figure 7b, which gives the broadside gain for a frequency interval of 0.1 GHz. The measured gains are consistent with the simulation, exceeding 0.2 dBi in the operating band of 220 MHz to 2.5 GHz. As shown in Figure 7b, the broadside gain of the proposed antenna is decreasing in the high frequency band. This is caused by the beam splitting near 10°, as shown in Figure 3c.

**Figure 8:**
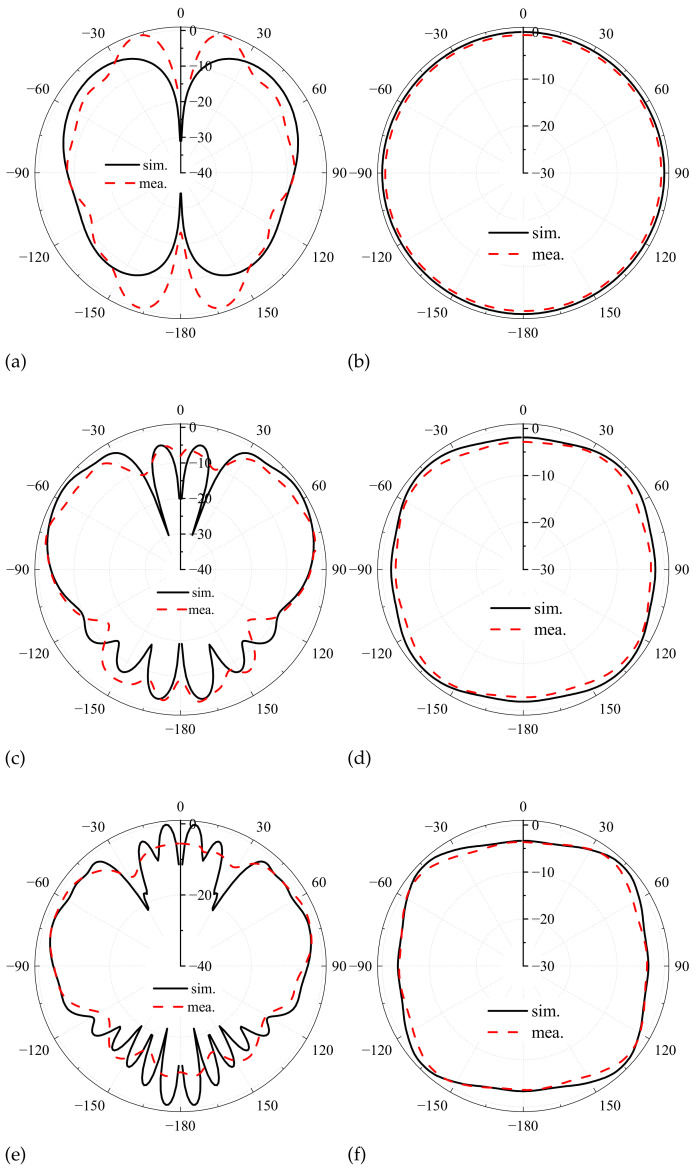
Normalized E- and H-plane patterns of antenna prototype at (**a**,**b**) 0.4 GHz; (**c**,**d**) 1.4 GHz; (**e**,**f**) 2.4 GHz.

Figure 8 shows the patterns of the antenna prototype in the E- and H-plane.The radiation patterns are broadside in the E-plane and omnidirectional in the H-plane. The proposed antenna has monopole-like radiation characteristics in the operating band. At 0.4 GHz, the radiation characteristics are consistent with Figure 3c. At 1.4 GHz, the E-plane pattern is slightly distorted, as shown in Figure 8d. This is due to the introduction of four shorting probes. This phenomenon is severe at 2.4 GHz, as shown in Figure 8f. The measured and the simulated E-plane patterns are inconsistent in the large angle, causing the machining error and the test error. Specifically, the prototype machining method is less accurate, and the near-field test has fewer sampling points in the E-plane.

The comparison of the proposed antenna with the cited wideband conical antenna is shown in Table 2. The plasma monocone antenna is designed using reconfigurable technique, but the gain of −5 dBi cannot meet the requirement [1]. A monocone antenna with lumped loading has a 9.3:1 band, but lumped elements reduce antenna efficiency [23,24]. Dielectric-loading can obtain a 7.3:1 impedance bandwidth [25,26] and adding a sleeve or slotting the patches obtain an operating band less than 2.3:1 [27,28]. All the bandwidth is not enough. Cone optimization can achieve a 26:1 bandwidth [29,30], but pattern consistency is not considered in the broadside. The discone antenna with pear-like cone has a bandwidth of 45.4:1 [14] and the stacked conical realizes the filling of the pattern null [19], but the height is not satisfactory. The proposed antenna overcomes the problem of high profile or pattern inconsistency for vehicle communication systems. Without increasing the antenna volume, two sleeves fill the pattern null in the broadside. The proposed antenna has a monopole-like pattern in a 11.36:1 band with a compact size.

## 4. Conclusions

A compact VHF/UHF ultrawideband discone antenna with consistent pattern is proposed in this article. Without increasing the antenna volume, two sleeves are inserted into the discone to obtain a monopole-like pattern over a 11.36:1 band. The worst pattern null is filled to −5.5 dB in the broadside. The proposed antenna demonstrates $|S_{11}|$ of below −10 dB, the gain exceeding 0.2 dBi in the operating band. A compact cylindrical volume of 0.227 $\lambda_{0}$ in diameter and 0.096 $\lambda_{0}$ in height are realized in the novel design. These properties make it a competitive candidate for VHF/UHF vehicle communication systems.

## Figures and Tables

**Table 2:** Comparison of wideband conical antennas.

Ref.	Design Principle	Im. BW	Null	Gain (dBi)	Size (D × H, $\lambda_{0}$)
[1]	electrically tuned plasma	30–100 or 100–512 MHz (5.1:1)	no	≥−**5**	0.05 × 0.1
[14]	polynomial function	0.4–18.2 GHz (45.5:1)	−25 @16 G	≥1	**0.20** × **0.283**
[19]	spherical-multipole optimized	3–20 GHz (6.67:1)	no	≥−0.7	**0.9** × **0.4**
[23]	lumped loading	0.118–1.1 GHz (9.3:1)	no	≥−**8**	0.24 × 0.06
[24]	composite loaded	80–600 MHz (7.5:1)	no	**/**	0.168 × 0.059
[25]	dielectric material	0.69–3.35 GHz (4.86:1)	−10 @3.2 G	≥1.3	0.115 × 0.092
[26]	dielectric loading	1.6–11.7 GHz (7.3:1)	no	≥ 3	**0.363** × **0.057**
[27]	adding shorted columns	0.84–1.69 GHz (2:1)	no	≥1.27	**0.44** × **0.07**
[28]	interdigital capacitor	1.66–3.95 GHz (2.38:1)	no	≥1	**0.672** × **0.081**
[29]	cone shape optimized	2.5–24 GHz (9.6:1)	−12 @9 G	≥2	0.192 × 0.05
[30]	supercurves	1.4–37.4 GHz (26:1)	−10 @30 G	≥2.5	**0.74** × **0.40**
[31]	four tapered slots	0.8–2.3 GHz (2.875:1)	no	≥2.9	**0.213** × **0.069**
This work	additional sleeves	0.22–2.5 GHz (11.36:1)	−5.5 @1.9 G	≥0.2	0.227 × 0.096

$\lambda_{0}$ is the wavelength in free space at lowest frequency.

## Data Availability

The data presented in this study are available on request from the corresponding author.

## References

[1] Wang C., Yuan B., Shi W., Mao J. (2020). Low-Profile Broadband Plasma Antenna for Naval Communications in VHF and UHF Bands. IEEE Trans. Antennas Propag..

[2] Choi J., Dagefu F.T., Sadler B.M. (2020). An Efficient Low-Profile Low-VHF Antenna for Small Unmanned Ground Vehicles. Proceedings of the 2020 14th European Conference on Antennas and Propagation (EuCAP).

[3] Choi J., Dagefu F.T. (2020). A Miniature High-Gain Low-VHF Antenna. Proceedings of the 2020 IEEE International Symposium on Antennas and Propagation and North American Radio Science Meeting.

[4] Park W.B., Park Y.M., Hwang K.C. (2020). Ferrite-Loaded Spidron Fractal Loop VHF Antenna for UAV Applications. Appl. Sci..

[5] Gao X., Ma Q., Gu Z., Cui W.Y., Liu C., Zhang J., Cui T.J. (2023). Programmable surface plasmonic neural networks for microwave detection and processing. Nat. Electron..

[6] Chen L., Ma Q., Luo S.S., Ye F.J., Cui H.Y., Cui T.J. (2022). Touch-Programmable Metasurface for Various Electromagnetic Manipulations and Encryptions. Small.

[7] Chung J.Y., Yih G. (2022). Low-Profile VHF Antenna Based on Quarter-Mode Substrate-Integrated Waveguide Structure. Appl. Sci..

[8] Islam M.A., Talukder M.K.H., Md Ahad S. Design and Analysis of Vehicle Mounted Whip Antennas for HF and VHF Communication. Proceedings of the 2023 4th International Conference for Emerging Technology (INCET).

[9] Ranjbar Nikkhah M., Dagefu F.T., Behdad N. (2020). Electrically Small Platform-Based Antennas for an Unmanned Ground Vehicle. IEEE Trans. Antennas Propag..

[10] Abdul-Rahman E., Aloi D.N. (2022). Design of a 5G Sub-6 GHz Vehicular Cellular Antenna Element with Consistent Radiation Pattern Using Characteristic Mode Analysis. Sensors.

[11] Jayaram M., Murugan S., Mohandass S., Anjaneyulu B.M. Design and Simulation of Ultra-Wideband antenna for Wireless applications. Proceedings of the 2022 3rd International Conference on Communication, Computing and Industry 4.0 (C2I4).

[12] Liu S., Liu J., Zhao L., Xie W., Hu N. Design of an Ultra- Wideband Discone Antenna. Proceedings of the 2022 International Conference on Microwave and Millimeter Wave Technology (ICMMT).

[13] Guo L., Min M., Che W., Yang W. (2019). A Novel Miniaturized Planar Ultra-Wideband Antenna. IEEE Access.

[14] Zhao Y., Wang W. Design of a Pear-Like Combined Discone Antenna with Super Wide Bandwidth. Proceedings of the 2021 IEEE 4th International Conference on Electronics Technology (ICET).

[15] Asthan R.S., Munir A. Design and Realization of A Wideband Quadratic Wire-shaped Discone Antenna. Proceedings of the 2023 Workshop on Microwave Theory and Technology in Wireless Communications (MTTW).

[16] Munir A., Sahlendar Asthan R., Andi Nurmantris D. Curvature Shape Utilization for Characteristics Enhancement of Skeletal Wire Discone Antenna. Proceedings of the 2023 IEEE-APS Topical Conference on Antennas and Propagation in Wireless Communications (APWC).

[17] Ng K.J., Islam M.T., Alevy A.M., Mansor M.F. (2020). Ultralow Profile, Low Passive Intermodulation, and Super-Wideband Ceiling Mount Antennas for Cellular and Public Safety Distributed Antenna Systems. Sensors.

[18] Munir A., Asthan R.S., Dwi Prananto H., Oktafiani F. Design and Characterization of PLA-based Wideband 3D-Printed Discone Antenna. Proceedings of the 2022 IEEE International Symposium on Antennas and Propagation and USNC-URSI Radio Science Meeting (AP-S/URSI).

[19] Rostomyan N., Ott A.T., Blech M.D., Brem R., Eisner C.J., Eibert T.F. (2015). A Balanced Impulse Radiating Omnidirectional Ultrawideband Stacked Biconical Antenna. IEEE Trans. Antennas Propag..

[20] Kim W., Shin G., Lee K.W., Mun B., Yoon I.J. (2022). A Wideband Monoconical Antenna for Airborne Applications with a Null-Filled Radiation Pattern. IEEE Antennas Wirel. Propag. Lett..

[21] Keum K.S., Park Y.M., Choi J.H. (2019). A Low-Profile Wideband Monocone Antenna Using Bent Shorting Strips. Appl. Sci..

[22] Balanis C.A. (2005). Antenna Theory: Analysis and Design.

[23] Zhang D., Wu Q. (2022). A miniaturised mono-cone antenna with top plate and composite lumped loading. Electron. Lett..

[24] Xu S.Y., Liu J., Hui Chen G. (2018). Design of a Composite Loaded UWB Miniaturized Vehicle-Mounted Antenna. Proceedings of the 2018 IEEE 18th International Conference on Communication Technology (ICCT).

[25] Omar A.A., Shen Z. (2019). A Compact and Wideband Vertically Polarized Monopole Antenna. IEEE Trans. Antennas Propag..

[26] Liu A., Lu Y. (2019). A Superwide Bandwidth Low-Profile Monocone Antenna With Dielectric Loading. IEEE Trans. Antennas Propag..

[27] Yang L., Xing Z., Xu S., Zhang D., Li Y. (2020). Design of a 3D Top-Loaded Low-Profile Sleeve Antenna for UAV Applications. IEEE Access.

[28] Wen S., Dong Y. (2020). A Low-Profile Wideband Antenna with Monopolelike Radiation Characteristics for 4G/5G Indoor Micro Base Station Application. IEEE Antennas Wirel. Propag. Lett..

[29] Yang D., Hu J., Liu S. (2018). A Low Profile UWB Antenna for WBAN Applications. IEEE Access.

[30] Gandomi M.H., Zarifi D. (2021). Design and Development of Ultra-Wideband 3-D Monopole Antennas Based on Supercurves. IEEE Trans. Antennas Propag..

[31] Nguyen-Trong N., Piotrowski A., Kaufmann T., Fumeaux C. (2016). Low-Profile Wideband Monopolar UHF Antennas for Integration Onto Vehicles and Helmets. IEEE Trans. Antennas Propag..

